# Supplementary material for: Ligand-induced conformational rearrangements regulate the switch between membrane-proximal and distal functions of Rho kinase 2
Source: Commun Biol. 2020 Nov 27;3:721. doi: 10.1038/s42003-020-01450-x (PMC7699638; doi:10.1038/s42003-020-01450-x)
Supplement: Supplementary file 1 — Supplementary Information [file 42003_2020_1450_MOESM1_ESM.pdf]

SUPPLEMENTARY FIGURES

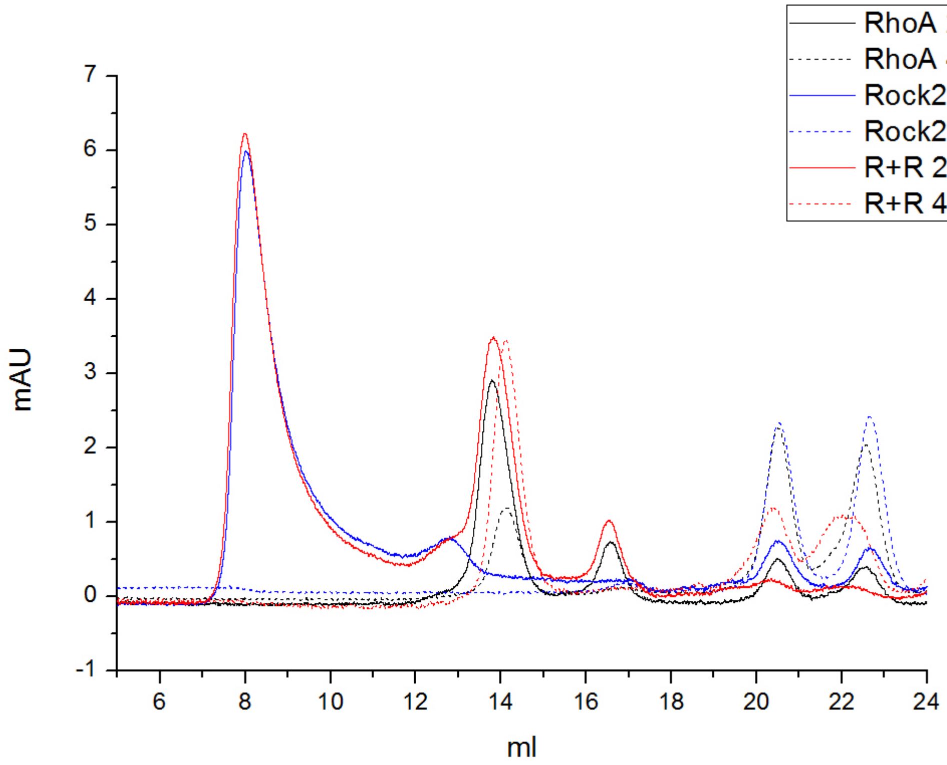

Supplementary figure 1: Raw data of size exclusion chromatography analysis of the ROCK2/RhoA/BODIPY-GTP complex on a Superose 12 column. 5 µM ROCK2, 5 µM RhoA and 5 µM BODIPY-GTP (red); 5 µM ROCK2 and 5 µM BODIPY-GTP (blue) and 5 µM RhoA and 5 µM BODIPY-GTP (black) was mixed and run and followed at 280 nm (solid line) and 488 nm (dotted line) absorbance. ROCK2 is detected at 8 ml; RhoA at 14 ml, while BODIPY-GTP exhibits a double peak at 21 and 23 ml. When BODIPY-GTP is used in a binary mix with ROCK2 (blue), no sign of complex formation is seen. In the binary mix with RhoA (black) a 488 nm absorbance peak at 14ml (RhoA size) appears indicating BODIPY-GTP binding to RhoA. Using the ternary mix (red) the 14 ml peak (488nm) significantly increases with the concomitant decrease of the double signal of the unbound BODIPY-GTP. Our results show that using SEC, the ROCK2/RhoA mix cannot be isolated and BODIPY-GTP easily dissociates from RhoA as well. Using the ternary mix between ROCK2/RhoA and BODIPY-GTP, ROCK2 stabilizes the interaction between RhoA and BODIPY-GTP reflecting the actual formation of a weak dynamic ternary complex.

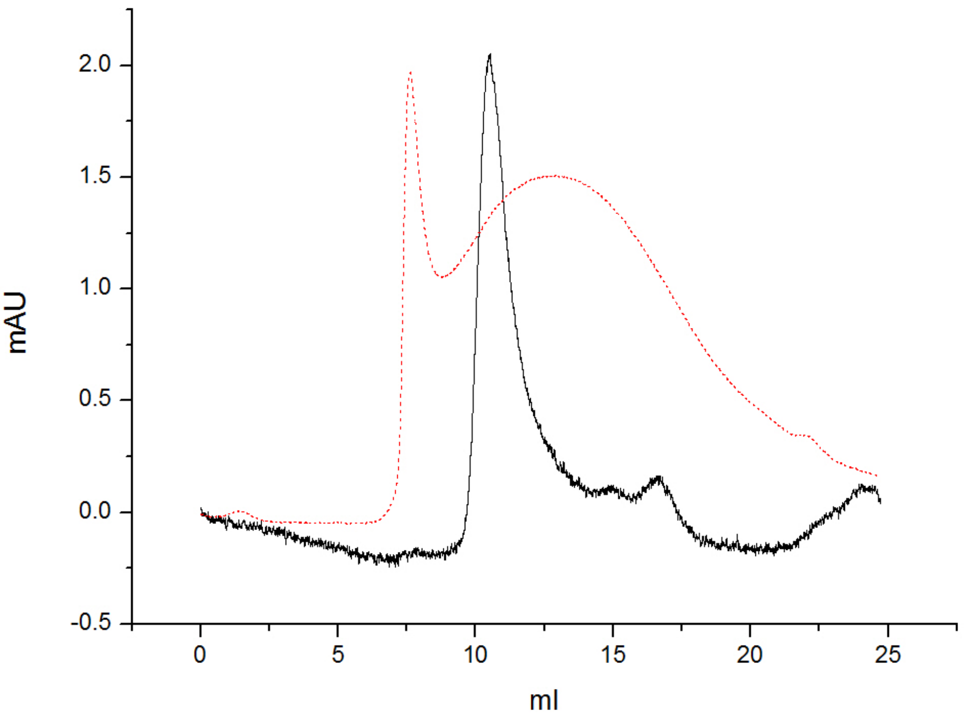

Supplementary figure 2: Size exlusion chromatography of MBP-ROCK2 on a Superose 6 column The ROCK2 sample peak (black solid line) appears at approx. 1.6 Mda size, however taken into account the elongated shape of the ROCK2 molecule it corresponds to the expected size The shape of the peak is nearly symmetric, and the retention volume (10.5ml) is significantly higher than the exclusion volume the first peak of Blue Dextran, red dashed line7.6ml) showing that ROCK2 is not forming aggregates.

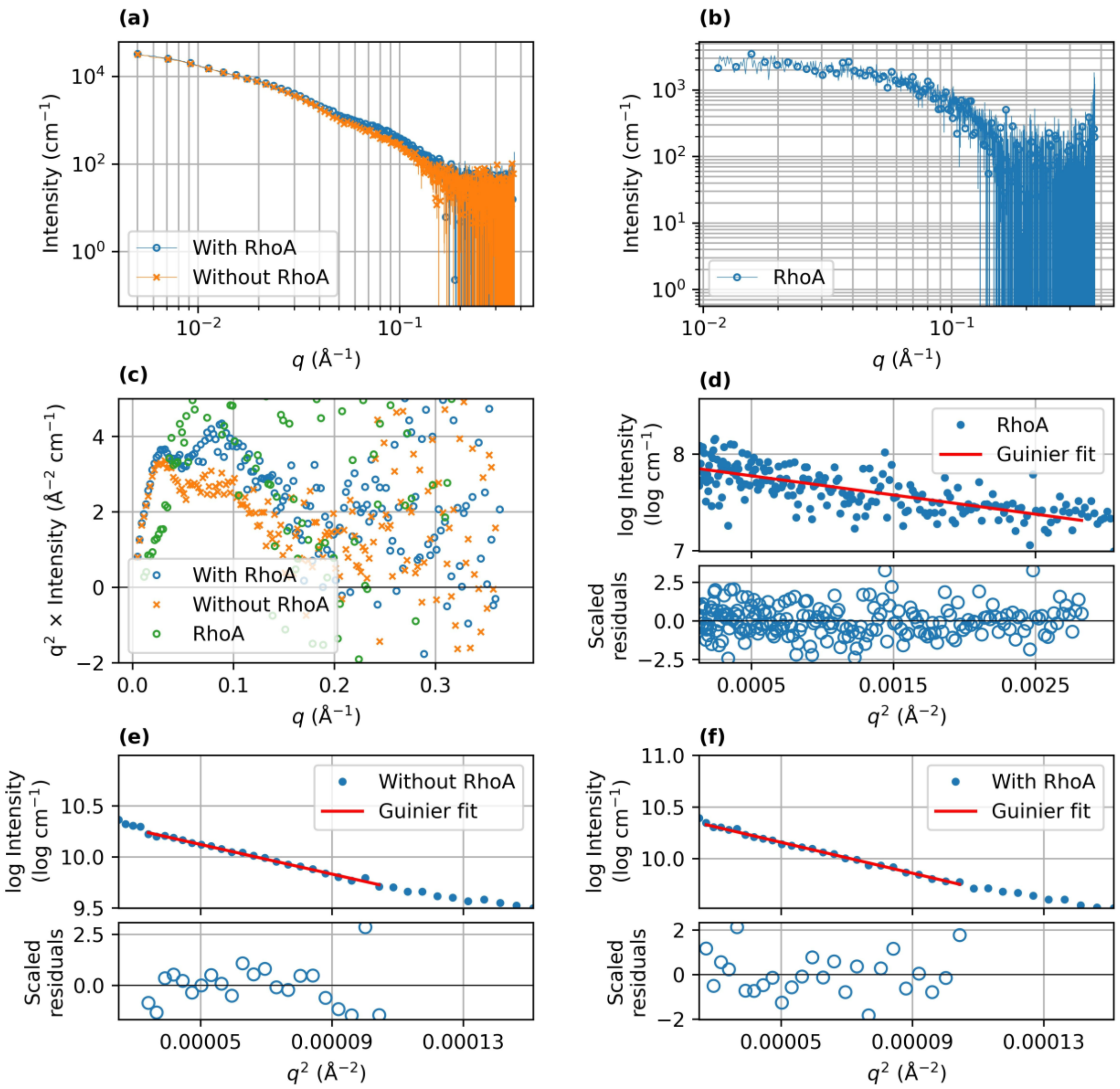

Supplementary figure 3: Original plots for ROCK2 in the absence and presence of RhoA and RhoA individually. Double logarithmic representation of scattering data (a)for ROCK2 in the absence (orange)and presence (blue) of RhoA; and RhoA individually (b) are shown. tThe Kratky plots for all three forms are plotted in one graph (c) and the Guinier plots for the RhoA individually(d); ROCK2 individually (e) and ROCK2 in the presence of Rho are shown (f). The Rg values are 2.34±0.13 nm for RhoA; 14.63±0.18 nm for ROCK2 and 15.09±0.16 nm for ROCK2 in the presence of RhoA.
